# Insight into the Key Points of Preeclampsia Pathophysiology: Uterine Artery Remodeling and the Role of MicroRNAs

**DOI:** 10.3390/ijms22063132

**Published:** 2021-03-19

**Authors:** Katarzyna Pankiewicz, Anna Fijałkowska, Tadeusz Issat, Tomasz M. Maciejewski

**Affiliations:** 1 Department of Obstetrics and Gynecology, Institute of Mother and Child in Warsaw, Kasprzaka 17a, 01-211 Warsaw, Poland; tadeusz.issat@imid.med.pl (T.I.); tomasz.maciejewski@imid.med.pl (T.M.M.); 2 Department of Cardiology, Institute of Mother and Child in Warsaw, Kasprzaka 17a, 01-211 Warsaw, Poland; anna.fijalkowska@imid.med.pl

**Keywords:** preeclampsia, two-stage model, uterine artery remodeling, microRNA

## Abstract

Preeclampsia affects about 3–8% of all pregnancies. It represents a complex and multifaceted syndrome with at least several potential pathways leading to the development of disease. The main dogma in preeclampsia is the two-stage model of disease. Stage 1 (placental stage) takes place in early pregnancy and is thought to be impaired placentation due to inadequate trophoblastic invasion of the maternal spiral arteries that leads to reduced placental perfusion and release of numerous biological factors causing endothelial damage and development of acute maternal syndrome with systemic multiorgan failure (stage 2—the onset of maternal clinical symptoms, maternal stage). Recently, in the light of the vast body of evidence, two-stage model of preeclampsia has been updated with a few novel pathways leading to clinical manifestation in the second part of pregnancy. This paper reviews current state of knowledge about pathophysiology of preeclampsia and places particular focus on the recent advances in understanding of uterine artery remodeling alterations, as well as the role of microRNAs in preeclampsia.

## 1. Introduction

Preeclampsia (PE) affects about 3–8% of all pregnancies. It is the main cause of perinatal morbidity and mortality in developed countries, responsible for about 16–18% of maternal deaths and about 40% of fetal and neonatal deaths [1]. PE is defined according to The International Society for the Study of Hypertension in Pregnancy (ISSHP) as the presence of a new-onset hypertension after 20 weeks’ gestation accompanied by proteinuria or evidence of maternal acute kidney injury, liver dysfunction, neurological features, hemolysis or thrombocytopenia, or fetal growth restriction (FGR). There are two types of PE: Early-onset disease developing before 34 weeks’ gestation and late-onset disease developing after 34 weeks’ gestation [2].

The knowledge about pathophysiology of PE is still improving. PE represents a complex and multifaceted syndrome with at least several potential pathways leading to the development of disease. It is a systemic condition with global endothelial dysfunction and multiorgan failure affecting both maternal and fetal health. Moreover, there is a vast body of evidence supporting a relationship between developing PE during pregnancy and future cardiovascular risk in mother as well as the offspring [3,4]. Women who developed PE have four-fold increased risk of heart failure, two-fold increased risk of coronary artery disease, two-fold increased risk of stroke, and two-fold increased risk of cardiovascular death later in life [5,6]. They also have increased risk of developing microalbuminuria and end-stage renal disease in the future [7,8,9]. Thanks to growing knowledge about the pathomechanism of PE, effective prophylaxis using acetylsalicylic acid is now available for women at high risk [10]. On the other hand, some points are still missing, and termination of pregnancy remains as the only effective therapy [2,11,12]. This narrative review aims to summarize recent advances in understanding of PE pathophysiology with particular emphasis on defective uterine artery remodeling and the role of microRNAs.

## 2. Two-Stage Model of Preeclampsia

The two-stage model of PE introduced by Redman in 1991 is the main dogma in PE pathophysiology [13]. Stage 1 (placental stage) is caused by impaired placentation due to inadequate trophoblastic invasion of the maternal spiral arteries that leads to reduced placental perfusion. Stage 2 (clinical, maternal stage) is postulated to be a consequence of stage 1: Release of numerous biological factors from ischemic placenta causes endothelial damage and development of acute maternal syndrome with systemic multiorgan failure [13,14]. At present, almost 30 years since the two-stage model of PE was developed, there is a strong evidence in support of a great number of factors connecting both disease stages. They include: Angiogenic and antiangiogenic factors (vascular endothelial growth factor VEGF, placental growth factor PlGF, soluble fms-like tyrosine kinase 1 sFlt-1, soluble endoglin sEng), hypoxia-induced factor 1α (HIF-1α), endothelin-1, syncytiotrophoblast microparticles (STBM), angiotensin II 1 receptor autoantibodies (AT1-AA), oxidative stress, and endoplasmic reticulum (ER) stress with unfolded protein response [15,16,17,18]. These factors are discussed intensively in other reviews [19,20,21,22,23,24,25,26].

In 2014, Redman and in 2019, Staff published an update to the two-stage model of PE. The most important finding is that there are at least two (potentially more) different pathways leading to stage 1: It is not only poor placentation with inadequate remodeling of maternal spiral arteries, but also intraplacental malperfusion due to mechanical restrictions causing syncytiotrophoblast (STB) stress. The first pathway reflects an “extrinsic cause” of placental dysfunction developing early in pregnancy and leading to early-onset PE accompanied very often by FGR. The second pathway depicts the situation when late in pregnancy the placenta outgrows uterine capacity and terminal villi are compressed impeding intervillous perfusion. This is called an “intrinsic cause” of placental dysfunction and is related to late-onset PE with normal fetal growth [27,28]. There is also another pathway that can lead to STB stress–a cellular senescence of ageing placenta. Upregulation of senescence factors (e.g., annexins) in preeclamptic placentas, as well as the shortening of telomere in placental cells during PE indicate the link between cellular senescence and PE [29].

**Figure 1:**
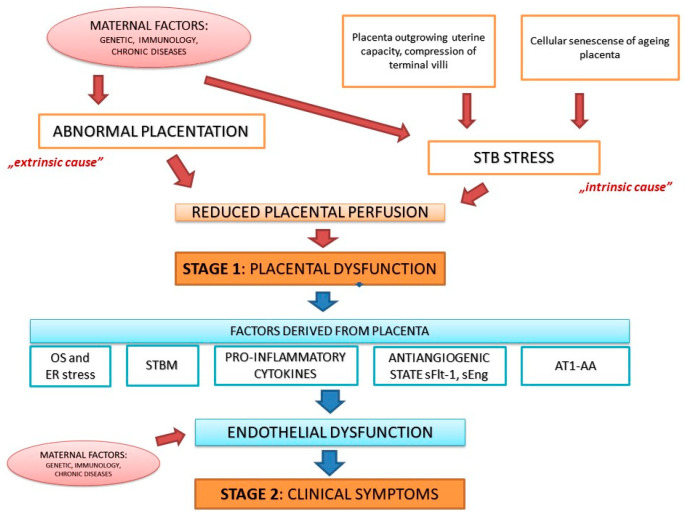
The updated two-stage model of preeclampsia (PE). STB–syncytiotrophoblast, OS–oxidative stress, ER–endoplasmic reticulum, STBM—syncytiotrophoblast microparticles, sFlt-1–soluble fms-like tyrosine kinase 1, sEng—soluble endoglin, AT1-AA—angiotensin II 1 receptor autoantibodies.

Another improvement to the two-stage PE model is inclusion of the findings that maternal factors may affect both stages of disease development. Maternal features including genetic predisposition, immunological factors, and chronic diseases may act via different pathways and impact placentation, placental size and function, as well as maternal susceptibility and responsiveness to factors shed by placental tissue accelerating the appearance of clinical symptoms in mother [28]. The updated two-stage model of PE is summarized in Figure 1.

## 3. Uterine Spiral Artery Remodeling

Uterine spiral artery remodeling takes place in early pregnancy when extravillous trophoblast (EVT) migrates to, invades, and replaces the vascular smooth muscle and endothelial cells of the spiral arteries. In consequence, these arteries change from high-resistance/low-capacity to low-resistance/high capacity vessels and ensure sufficient blood flow for developing both placenta and fetus. Altered uterine artery remodeling is the focal point of PE pathophysiology [30].

Normal uterine artery remodeling is divided into two phases. First is the decidua-associated (or trophoblast-independent) remodeling conducted by decidual natural killer (NK) cells and macrophages that infiltrate vascular smooth muscle, partially through the activation of matrix metalloproteinases (MMP). The second stage is the trophoblast-dependent remodeling achieved by interstitial trophoblast that migrate towards the spiral arteries, replace the endothelium, and further disrupt the arterial muscular lining from inside the arterial lumen [31]. Both in vitro and clinical studies have demonstrated that a large number of factors have the ability to alter migratory and invasive capacity of trophoblast cells.

Uterine NKs are reported to be less cytotoxic and produce more cytokines than other NK types. A crosstalk between NKs, macrophages, and trophoblasts is crucial for maintaining normal spiral arteries remodeling. NKs recognizes trophoblast through killer-cell immunoglobulin-like receptors (KIRs) [32]. Patients with PE express the inhibitory, not the stimulatory KIR receptors and women with KIR AA genotype (related to inhibitory KIR2DL-1, -2, -3, and -5) are at increased risk of PE, whereas KIR B centromeric region, present in African women, protects them against the development of disease [30,31,33]. During PE there is also an increased activation of nuclear factor kappa B (NF-κB). Activation of uterine NKs by trophoblast leads to the release of proinflammatory cytokines in the NF-κB-dependent mechanism. NF-κB influences not only the expression of cytokines, but also KIRs, angiogenic factors, macrophages activation, and secretion of MMPs. The role of NF-κB in uterine artery remodeling has been recently reviewed by Socha et al. [34]. There is also evidence for important role of MMP-2 and MMP-9 in the development of PE. Timokhina et al. demonstrated decreased blood levels of MMP-9 in patients with early-onset PE in comparison to late-onset disease, suggesting the participation of MMP-9 in the spiral artery transformation. They also revealed significantly increased plasma concentrations of MMP-2, both in early-onset and late-onset PE, confirming the participation of MMP-2 in endothelial dysfunction in the second stage of PE [35].

Available studies indicate an important role of complement-dependent pathway in the development of PE. Endovascular trophoblasts are directly exposed to maternal blood containing complement components; however, they are not eliminated by complement-dependent cytotoxicity. Using the human EVT-like cell line Swan71, Ueda et al. demonstrated that the expression of CD59 may ensure some protection against maternal complement attack [36]. The analysis of maternal proteomes by Youssef et al. confirmed the involvement of activation of complement and coagulation cascades in the pathogenesis of PE. They found five significantly enriched pathways with excessive activation of complement and coagulation and then validated these results by assessing the deposits of C5b-9 complement complex on endothelial cells exposed to activated plasma from women with severe early-onset PE and uncomplicated pregnancy. C5b-9 deposits were significantly higher in patients with PE [37]. Lokki et al. investigated genetic variants for complement receptors CR3 and CR4 in PE and found that CR3 variant M441K related to increased adhesion to iC3b was the most significant variant in women with PE [38].

Regulation of uterine artery remodeling is also strictly related to antiangiogenic state. The imbalance between angio- and antiangiogenic factors, with the shift in favor of the latter, is currently one of the best documented elements of PE pathophysiology [19,22]. However, recent studies revealed interesting findings concerning sEng indicating its much more relevant role in PE than initially proposed. sEng is a cell surface co-receptor for TGFβ1 and TGFβ3 that influences vascular endothelial function. Significantly higher serum sEng levels are found in patients with PE [39]. Gallardo-Vara et al. found that sEng induces the expression of bone morphogenic protein 4 (BMP4) in endothelial cells. In animal model, after crossing female wild type with male sEng+ mice, PE-like syndrome appeared coinciding with the elevation in plasma levels of BMP4. Moreover, sEng-induced hypertension was absent in the presence of the BMP4 inhibitor–noggin, suggesting that BMP4 is a downstream regulator of sEng. In human, serum levels of sEng and BMP4 were positively correlated in pregnant women [40]. Perez-Roque et al. proposed a new model of sEng role in PE. They demonstrated that high levels of sEng are directly involved in the development of maternal symptoms, but sEng can also induce placental alterations (inflammation, oxidative stress, and hypoxia) due to pseudovasculogenesis and a diminished proliferative and invasive capacity of trophoblasts [41]. Additionally, two new binding partners for sEng have been recently identified: Galectin-3 and tripartite motif-containing protein 21 (TRIM21) [42].

Galectin-3 is a member of β-galactoside-binding lectins expressed in human tissues including epithelial cells, endothelial cells, all types of immune cells and it serves important functions in numerous biological processes, such as inflammatory response and fibrosis, intercellular adhesion, angiogenesis, cell differentiation and apoptosis [43]. Galectin-3 overexpression is associated with myocardial fibrosis and leads to cardiac dysfunction and development of heart failure [44,45]. The body of evidence for galectin-3 involvement in PE pathophysiology is now rapidly increasing. Experimental research of human placental cell line BeWo confirmed galectin-3 is one of the hypoxia-induced factors [46]. In proteomic analysis of blood samples obtained from patients with high risk of developing PE during first trimester upregulation of 10 proteins in women who developed PE later in pregnancy in comparison to those in uncomplicated pregnancy has been identified. One of these proteins was galectin-3 binding protein [47]. Sattar et al. demonstrated elevated serum galectin-3 levels in patients with PE that correlated with insulin resistance and dyslipidemia [48]. Ruikar et al. revealed the increased expression of annexin A1 and galectin-3 in placental tissue of preeclamptic women, suggesting the role of these proteins in PE pathophysiology by participating in a systemic inflammatory response [49]. Furthermore, the development of FGR is accompanied by an altered pattern of circulating galectin-3 levels and galectin-3 deficiency in mouse pregnancy leading to placental insufficiency and the subsequent development of FGR [50]. It is well established that galectin-3 stimulates angiogenesis through the VEGF receptor dependent pathway and may also inhibit apoptosis in different types of cells. These galectin-3 functions may be pivotal in the pathophysiology of PE [51,52].

Other galectins are also involved in the development of PE. Than et al. identified placental expression of a gene cluster on Chromosome 19 that codes for a subfamily of galectins, including galectin-13, -14, and -16. These galectins induce apoptosis of activated T lymphocytes and contribute to a disturbed maternal immune balance in early pregnancy [53,54]. Placental expression of galectin-13 and -14 is downregulated in preterm PE and associated with FGR [54]. Moreover, galectin-13 secretion from the STB into the maternal circulation and decreased galectin-13 blood concentration in the first trimester was found in women who subsequently develop early-onset PE [55,56].

Another recently reported factor that might be involved in the development of PE by influencing placental angiogenesis is fractalkine (CX3CL1). CX3CL1 is a chemotactic factor, expressed in placental tissue, mainly in STB and shed to maternal circulation. CX3CL1 receptor is present in NKs, macrophages, and T lymphocytes. CX3CL1 is able to induce angiogenesis via HIF-1α/VEGF pathway, as well as to stimulate integrin-dependent trophoblast migration [57,58,59]. Placental CX3CL1 have been reported to be upregulated in early-onset PE and also to be deregulated by angiotensin II. Thus, it may contribute to a pro-inflammatory trophoblast-monocyte interaction [60,61]. Szewczyk et al. reported significantly higher CX3CL1 serum levels, as well as its placental expression in patients with PE and their negative correlation with the vascular/extravascular tissue index. The authors suggested that significant underdevelopment of placental vascular network in PE is associated with the alteration in CX3CL1 expression and distribution [62].

## 4. MicroRNAs

MicroRNAs (miRNAs) are small (consisting of about 22 nucleotides), single-stranded, noncoding RNA molecules. They do not translate proteins, however they can regulate gene expression and miRNAs regulatory networks play important role in many pathophysiological processes, such as cell proliferation and adhesion, angiogenesis, and immune cell development [63]. Available research suggest that during pregnancy miRNAs may influence and regulate trophoblast cell invasion and migration, angiogenesis (by regulating expression of angiogenic and antiangiogenic factors, including VEGF, sFlt-1, HIF-1α), as well as mesenchymal stem cell function [64,65]. Moreover, cardiovascular and cerebrovascular disease associated miRNAs are dysregulated in pregnancies complicated by PE and FGR and they may play a pivotal role in cardiac adaptation to pregnancy and gestational hypertension [66,67,68]. Specific patterns of miRNA have been detected in the placenta and maternal circulation in patients with PE [64,69,70].

Huang et al. revealed that miRNA-181a-5p is overexpressed in human preeclamptic placentas compared with healthy controls. They also demonstrated that miRNA-181a-5p may trigger antiproliferation, inhibit cell cycle progression, induce apoptosis, and suppress trophoblast invasion by inhibiting the activity of metalloproteinases 2 and 9 [71]. In other study it was found that miRNA-181a-5p could suppress invasion and migration of HTR-8/SVneo cells by directly targeting insulin-like growth factor 2 mRNA-binding protein 2 (IGF2BP2) [72]. Brkic et al. investigated a role of miRNA-218-5p in the development of PE and demonstrated that miRNA-218-5p was decreased in preeclamptic placentas compared with healthy controls. In the first trimester the expression of miRNA-218-5p is upregulated and induces differentiation of extravillous trophoblast (EVT) into endovascular type (enEVT). MiRNA-218-5p accelerates spiral artery remodeling and this effect is mediated by the suppression of transforming growth factor β2 (TGF-β2) signaling. Because miRNA-218-5p is downregulated in preeclamptic placentas and serum TGF-β2 is increased in PE patients, the authors of this study suggested that downregulation of miRNA-218-5p may contribute to the development of PE [73]. TGFβ pathway is known as a negative regulator of trophoblast invasion. In HTR-8/SVneo cells and placental explants, TGFβ inhibited EVT invasion and additionally incubation with TGF-β2 resulted in downregulation of enEVT markers in trophoblast cell line. Moreover, silencing of TGFB2 mimicked the miR-218-5p phenotype [73,74]. Hu et al. reported that the expression of miRNA-144-3p was decreased in preeclamptic placentas and it was negatively correlated with placental expression of cyclooxygenase-2 (Cox-2), an enzyme of prostaglandin synthesis, that may play an important role in the development of PE. The association between miRNA-144-3p and Cox-2 was also confirmed in vitro [75]. Guo et al. demonstrated that miRNA-133a-3p can relieve the oxidative stress-induced apoptosis in the trophoblast cells through the BACH1/Nrf2/HO-1 signaling pathway [76]. On the other hand, other research revealed that miRNA-133a-3p overexpression in umbilical cord blood was strictly related to the centralization of fetal circulation in patients with PE [77].

Many placental miRNAs with high level of expression are encoded by the C19MC-cluster located in chromosome 19. This is the largest human miRNA gene cluster discovered to date and it is expressed mainly in the placental tissue (primate-specific cluster). It consists of 46 miRNAs and is derived from paternally inherited chromosomes. Although its exact role is not yet known, it is undoubtedly very important for the maintenance of normal placental function because of its high level of expression [78].

Studies on miRNAs demonstrated the existence of placental miRNAs in maternal plasma, which modulate gene expression in the maternal compartment. One of the best-known miRNA involved in PE pathophysiology is miR-210, which can be found upregulated both in the placenta and maternal circulation of preeclamptic women [64,69,79,80]. MiRNA-210 is considered as hypoxia-associated and regulates trophoblast invasion, i.a. via targeting potassium channel modulatory factor-1 signaling in the human placenta [81]. More than sixty genes have been experimentally indicated as targets of miRNA-210, including mitogen activated protein kinase (MAPK), iron-sulfur scaffold homologue (ISCU), 17-beta-hydroxysteroid dehydrogenase, ephrin-A3, and homeobox-A9 [82,83]. Youssef et al. found 5-fold increase in the expression of miRNA-210 in serum of preeclamptic patients in comparison to healthy controls and revealed that miRNA-210 may serve as a predictor of the severity of PE. They also demonstrated 2-fold increase in the expression of miRNA-155 in serum of preeclamptic women [84]. MiRNA-155 overexpression contributes to the development of PE by inhibiting cysteine-rich angiogenic inducer 61 (CYR61)—factor involved in regulation of angiogenesis in early pregnancy and also by reducing the stability of CYR61 mRNA leading to local ischemia and oxidative stress [85]. MiRNA-155 is considered as an inflammatory-related miRNA, as it can be significantly up-regulated by tumor necrosis factor-α [86]. MiRNA-155 is also strictly related to interleukin-17A and can regulate nuclear factor NF-kB [87]. Ayoub et al. demonstrated that miRNA-155rs767649 polymorphism is associated with increased risk of PE [86].

Zhong et al. identified the differential expression profiles of microRNA in the plasma between patients with PE and normal pregnancies using microarray methods. They demonstrated that 3 miRNAs were upregulated and 26 miRNAs were downregulated in the plasma of preeclamptic women. The upregulated miRNAs were hsa-miRNA-1304-5p, hsa-miRNA-320a, and hsa-miRNA-5002-5p. The five most downregulated miRNAs were hsa-miRNA-188-3p, hsa-miRNA-211-5p, hiv1-miRNA-TAR-3p, hsa-miRNA-4432, and hsa-miRNA-4498. Functional analysis of differentially expressed miRNA in this study revealed that upregulated miRNAs were involved in the cell proliferation and migration, whereas downregulated miRNA in immune regulation, vascular development, and cancer pathology (cell cycle progression) [88]. Similar analysis was performed by Lip et al., but it concerned early-onset PE. The plasma concentration of 26 miRNAs were different in women with PE in comparison to healthy controls. Transfection of endothelial cells with the 3 miRNAs with the greatest fold change (miRNA-574-p, miRNA-1972, and miRNA-4793-3p) demonstrated that miRNA-574-p and miRNA-1972 decrease the proliferation and migration, as well as the tube-formation capacity of endothelial cells. Authors concluded that these 2 miRNAs significantly influenced endothelial angiogenic function, contributing to the development of PE [89].

There is evidence that not only whole plasma, but also exosomal miRNA may have important role in the pathophysiology of PE. Exosomes, one of the extracellular vesicles (EVs), containing proteins, lipids, and miRNAs, can be secreted by different cells, including red blood cells, fibroblasts, endothelial, and trophoblast cells. After secretion from cells, EVs may modulate the activity of adjacent cells or travel to distal regions transmitting unique signals, serving as a non-hormonal way of intercellular communication [82,83]. During pregnancy, this way of communication is very important in transmitting signals between mother and fetus [90]. Li et al. found that the concentration of plasma exosomes from PE and FGR was 1,5-fold higher compared to healthy controls. Moreover, they demonstrated that seven miRNAs were differentially expressed in exosomes from women with PE, but only one of them was significantly different in whole plasma miRNA analysis [91]. Noteworthy, not all miRNAs are expressed in exosomes. In the study performed by Devor et al., one-third of the 368 miRNAs profiled were not expressed in exosomes. However, among miRNAs that were expressed in exosomes, a total of eight were found to differ significantly in their expression between women who developed PE and those who did not. Five of them were overexpressed (miRNA-134, miRNA-196b, miRNA-376c, miRNA-486-3p, and miRNA-590-5p) and three were downregulated (miRNA-302c, miRNA-346, and miRNA-618) [92]. MiRNA-302c is a member of evolutionarily ancient miRNA family found on chromosome 4, targeting cyclin D1 and AKT1 expressed in embryonic stem cells. The loss of AKT activity in placental tissue of preeclamptic women significantly increase circulating levels of sEng [92,93]. MiRNA-346 expression is induced in response to ER stress through a two-step mechanism: Activation of inositol-requiring enzyme 1 and then X-box binding protein 1 [92,94].

Different types of miRNA, thanks to their long half-life and high stability in extracellular fluids, such as serum, plasma, and urine, may be used as a diagnostic and early screening tool for PE [65,78]. Moreover, improved knowledge about miRNA involved in the development of PE can help to find targets for potential therapy. Kim et al. demonstrated that aspirin prevents TNF-α-induced endothelial cell dysfunction by regulating the NF-kB-dependent miRNA-155/eNOS pathway [95]. Yuan et al. revealed that traditional Chinese plant medicine–ligustrazine induce miRNA-16-5p inhibition and upregulates IGF-2 expression leading to impediment in PE progression [96].

Certain types of miRNA can also be the link between pregnancy-related complications and future risk of cardiovascular diseases. Hromadnikova et al. performed postpartum profiling of miRNA involved in the pathogenesis of cardiovascular and cerebrovascular diseases in women exposed to gestational hypertension, early- and late-onset PE and FGR. They found that epigenetic profile of miRNAs has been changing with advancing time, since several types of miRNA, which were upregulated postpartum in women exposed to hypertensive disorders of pregnancy were not observed to be dysregulated during the clinical manifestation of the disease [97].

The most common miRNA involved in PE with their target and function are presented in Table 1.

## 5. Other Mechanisms of Regulation of Gene Expression

MiRNAs are part of one of the most important mechanisms of regulation of gene expression during PE-epigenetics. Environmental factors can cause change in gene expression without hereditary change in DNA sequence including DNA methylation, histone modification, and noncoding RNAs: miRNAs, long noncoding RNAs (lncRNAs), and messenger RNAs (mRNAs) [98].

DNA methylation is the covalent addition of a methyl group to a cytosine in cytosine-phopsho-guanine (CpG) dinucleotides [98]. DNA methylation abnormalities in PE have been analyzed in the placenta, but also in circulating maternal blood cells, cell-free DNA, and maternal endothelial cells [99]. There is a large number of genes that are differentially methylated in PE, including i.a. IGF-1, IGF-2, HSD11B2, TET2 (involved in demethylation of MMPs), CXCL1, ALCAM, AGT, VEGF, and TNF. It has been discussed in detail in other reviews [98,99,100]. Recently, Dave at al. demonstrated hypomethylation of angiogenic factors—PlGF and sFlt-1 in the placenta [101].

Histone modification is activating or suppressing the expression of genes by enzymes, including acetylation, phosphorylation, sumoylation, methylation, ubiquitination, deamination, and Adenosine diphosphate (ADP)-ribosylation [98]. Recent studies demonstrated that both chronic placental ischemia and acute hypoxia have great impact on histone modifications during PE [102]. However, current knowledge concerning the role of histone modification in placentation is based mainly on animal studies and requires further research [98,99]. One of the crucial examples of histone modification in PE is expression of chymas–angiotensin II-generating enzyme in maternal endothelial cells. Chymas increase angiotensin II production by histone deacetylase inhibition [103]. In relation to the trophoblast invasive capacity, differential expression of MMPs (MMP-2 and MMP-9) and their tissue inhibitors (TIMPs) is associated to histone H3K9/27me3 [104].

LncRNAs are RNAs greater than 200 nucleotides in length, that do not translate proteins. The function of lncRNAs during PE and placental development is poorly understood. However, it has been demonstrated that they are involved in proliferation, invasion, and migration of trophoblasts [98,99]. Medina-Bastidas et al. performed gene expression microarray analysis on placental tissue obtained at delivery from PE and FGR patients and demonstrated that 36 lncRNAs were upregulated and 98 were downregulated in FGR and 9 lncRNAs were differentially expressed in PE and FGR group in comparison to uncomplicated pregnancy. Functionally these lncRNAs were involved in cytokine signaling pathways, protein modification, and regulation of JAK-STAT cascade [105]. Li et al. demonstrated that urate (hydroxyiso-) hydrolase, pseudogene (URAHP), which is a lncRNA, had significantly higher expression in PE placentae and therefore may be associated with PE [106]. Other lncRNAs that may be involved in PE pathophysiology are: IGF2/H19, MEG3, SPRY4-ITI, HOTAIR, MALATI, FLT1P1 and CEACAMP8 [107].

Among mechanisms of regulation of gene expression alternative splicing may be also of importance in the pathophysiology of PE. It has been suggested that an alternative exon splicing event that will generate a protein encoded by the first 13–15 exons of the FLT1 is a cause of excessive sFLt-1 production in PE [108]. Ruano et al. performed alternative splicing analysis on a global scale in the placenta in patients with PE and FGR and demonstrated that in PE 1060 and in FGR 1409 genes were affected with this event. They found that these alterations are potentially genetically based and identified four chromosomal regions that impact the splicing of genes in the placenta [109].

## 6. Summary

This review summarizes current knowledge about factors and processes involved in the development of PE. It is focused on recently reported factors involved in defective uterine artery remodeling—a key point in PE pathophysiology, as well as the role of miRNAs.

Although over the last 30 years knowledge about PE underlying pathophysiology has significantly improved, there is still a need for further research that will hopefully lead to successful therapy. The full explanation of molecular mechanism involved in various stages of the disease can help identify proper, specific targets for effective treatment. Because of increasing incidence of late PE complications, occurring in patient’s later life, many years after pregnancy, there is also a need for wide ranging research aiming to identify link factors and work out effective and detailed preventive strategies for these patients. Studies on the role of miRNAs seems to be particularly important as they can allow the application of individual, tailor-made preventive and therapeutic strategies.

## Figures and Tables

**Table 1:** The most common miRNA involved in PE [64,65,70,78].

miRNA Type	Expression	Localization	Target	Function
miRNA-181a-5p	upregulated	placenta	IGF2BP2, MMP2 and MMP9	Inhibit cell cycle Induce apoptosis inhibit trophoblast invasion
miRNA-218-5p	downregulated	placenta	TGFβ2	Induce differentiation of EVT into enEVT Accelerate spiral artery remodelling
miRNA-144-3p	downregulated	placenta	Cox-2	Prostaglandin synthesis and vasoconstriction
miRNA-133a-3p	upregulated	umbilical cord blood	BACH1	Inhibit the oxidative stress-induced apoptosis in the trophoblast cells
miRNA-210	upregulated	placenta and blood	MAPK, ISCU, HSD17, EFNA, HOXA9	Inhibit cell migration and invasion Inflammation-related pathway
miRNA-155	upregulated	placenta	CYR61, IL-17A, NF-kB, VEGF, cyclin D1	Inhibit cell proliferation and invasion Angiogenesis regulation Inflammation-related pathway
miRNA-574-p miRNA-1972	upregulated	blood	MKI67, SLC31A1, RSAD2, CXCL10	Inhibit cell proliferation and migration Inhibit the tube-formation capacity of endothelial cells
miRNA-302c	downregulated	exosomes	AKT1, cyclin D1	Angiogenesis regulation
miRNA-346	downregulated	exosomes	TAP1	ER stress
miRNA-223	downregulated	placenta	STAT3, FOXO1	Trophoblast invasion Apoptosis Oxidative stress
miRNA-148a miRNA-152	upregulated	placenta	HLA-G	Trophoblast invasion Function of T lymphocytes and NK cells in the decidua

IGF2BP2—Insuline growth factor 2 binding protein 2; MMP2 and 9—Metalloproteinase 2 and 9; TGF-β2—Transforming growth factor β; Cox-2—Cyclooxygenase 2; BACH1—BTB and CNC homology 1, basic leucine zipper transcription factor 1; MAPK—Mitogen activated protein kinase; ISCU—Iron-sulfur scaffold homologue; HSD17—17-beta-hydroxysteroid dehydrogenase; EFNA—Ephrin-A3; HOXA9—Homeobox-A9; CYR 61—Cysteine-rich angiogenic inducer 61; IL-17A—Interleukin 17A; NF-kB—Nuclear factor kappa-light-chain-enhancer of activated B cells; VEGF—Vascular endothelial growth factor; MKI67—Marker Of Proliferation Ki-67; SLC31A1—Solute carrier family 31 member 1; RSAD2—Radical SAM domain-containing 2 (viperin); CXCL10—C-X-C motif chemokine ligand 10; AKT1—AKT serine/threonine kinase 1; TAP1—ER antigen peptide transporter 1; STAT3—Signal transducer and activator of transcription 3; FOXO1—Forkhead box protein O1; HLA-G—Human leukocyte antigen G.

## Data Availability

No new data were created or analyzed in this study. Data sharing is not applicable to this article.
